# CD200R/CD200 Inhibits Osteoclastogenesis: New Mechanism of Osteoclast Control by Mesenchymal Stem Cells in Human

**DOI:** 10.1371/journal.pone.0072831

**Published:** 2013-08-05

**Authors:** Audrey Varin, Charalampos Pontikoglou, Elodie Labat, Frédéric Deschaseaux, Luc Sensebé

**Affiliations:** 1 STROMALab, UMR Univ. P. Sabatier/CNRS 5273, INSERM U1031, Toulouse, France; 2 Etablissement Français du Sang Pyrénées- Méditerranée, Toulouse, France; 3 Department of Hematology, University of Crete, School of Medicine, Heraklion, Crete, Greece; French Blood Institute, France

## Abstract

Bone homeostasis is maintained by the balance between bone-forming osteoblasts and bone-degrading osteoclasts. Osteoblasts have a mesenchymal origin whereas osteoclasts belong to the myeloid lineage. Osteoclast and osteoblast communication occurs through soluble factors secretion, cell-bone interaction and cell–cell contact, which modulate their activities. CD200 is an immunoglobulin superfamilly member expressed on various types of cells including mesenchymal stem cells (MSCs). CD200 receptor (CD200R) is expressed on myeloid cells such as monocytes/macrophages. We assume that CD200 could be a new molecule involved in the control of osteoclastogenesis and could play a role in MSC–osteoclast communication in humans. In this study, we demonstrated that soluble CD200 inhibited the differentiation of osteoclast precursors as well as their maturation in bone-resorbing cells *in vitro*. Soluble CD200 did not modify the monocyte phenotype but inhibited the receptor activator of nuclear factor kappa-B ligand (RANKL) signaling pathway as well as the gene expression of osteoclast markers such as osteoclast-associated receptor (OSCAR) and nuclear factor of activated T cells cytoplasmic 1 (NFATc1). Moreover, MSCs inhibited osteoclast formation, which depended on cell–cell contact and was associated with CD200 expression on the MSC surface. Our results clearly demonstrate that MSCs, through the expression of CD200, play a major role in the regulation of bone resorption and bone physiology and that the CD200-CD200R couple could be a new target to control bone diseases.

## Introduction

Bone is a highly dynamic tissue that is constantly remodeled by a process involving bone resorption by osteoclasts and bone formation by osteoblasts. An intricate balance between the activities of these two cell types maintains bone’s physiology.

Osteoclasts originate from hematopoietic lineage cells derived from bone-marrow myeloid precursors or circulating monocytes. These myeloid precursors fuse at the bone surface to form multinucleated osteoclasts that efficiently resorb bone. The first step of osteoclastogenesis is the formation of osteoclast precursors that depend on macrophage colony-stimulating factor (M-CSF) and express a high level of receptor activator of NF-κB (RANK). Mature osteoclasts are induced by the activation of the RANK receptor by RANK ligand (RANKL) [1]. Binding of RANKL induces receptor oligomerization and recruitment of signaling adaptor molecules such as tumor necrosis factor receptor–associated factor (TRAF) and more specifically TRAF6 [2–4]. Downstream of TRAF6, RANKL activates different signaling pathways, including mitogen-activated protein kinases (MAPKs) such as c-Jun terminal kinase, extracellular signal-regulated kinase (ERK), and p38, and then a cascade of transcription factors, including NF-κB, c-Fos, Fra-1, and nuclear factor of activated T cells cytoplasmic 1 (NFATc1). Activation of NFATc1, the master gene of the osteoclast differentiation [5], leads to the expression of genes involved in osteoclast activation such as tartrate-resistant acid phosphatase (trap), Osteoclast-associated receptor (*oscar*) and cathepsin K (*ctsk*) [6,7]. Osteoblasts express both M-CSF and RANKL and modulate osteoclast activity. They also express osteoprotegerin (OPG), which acts as a decoy receptor for RANK/RANKL binding. OPG binds to RANKL and prevents osteoclast differentiation and activation [8]. The balance between bone resorption and bone formation is mediated by soluble factors, but osteoblasts and osteoclasts can directly interact for regulation of bone homeostasis.

The role of CD200 in osteoclastogenesis was studied in mice, with conflicting results. Endogenous overexpression of CD200 suppressed osteoclast formation [9], whereas CD200-deficient osteoclasts were defective in multinucleation, associated with an inhibition in signaling downstream of RANK, essential for osteoclastogenesis [10]. The receptor of CD200 (CD200R) is a type 1 transmembrane glycoprotein of the Ig superfamily present on most leukocytes, especially the myeloid lineage [11,12], and mediates inhibitory signaling in myeloid cells [13,14]. Therefore, CD200 modulated differentiation of dendritic cells which induce regulatory T cell maturation [15,16].

Osteoblasts are derived from mesenchymal stem cells (MSCs); a recent study identified CD200 as a new marker for MSCs and an efficient marker to purify native MSCs [17].

We postulated that human CD200 can inhibit osteoclastogenesis and that MSCs can modulate osteoclast formation through the CD200–CD200R interaction. We demonstrated that CD200 inhibited the formation of multinuclear TRAP-positive cells and their bone-degradation capacities. Inhibition of osteoclast formation was associated with the inhibition of downstream signaling of RANK. Finally, we demonstrated that MSCs, via CD200, inhibited osteoclast formation. MSCs may be important players in bone mass regulation and the CD200–CD200R interaction may play a major role in the control of osteoclastogenesis for a novel therapeutic strategy to control bone disease.

## Materials and Methods

### Ethics statement

Bone marrow was aspirated from the posterior iliac crest of adults undergoing orthopedic surgery after approval of the Medical Ethics Committee of Tours (named “Comité de Protection des Personnes Tours – CPP Région Centre [Ouest-1]”) and in accordance with their guidelines. A written informed consent is signed by the patients for the use of their samples. 

### Reagents and antibodies

Recombinant human M-CSF and soluble recombinant human RANKL were purchased from Peprotech (London, UK). Anti ERK, phospho-ERK, p38, phospho-p38, and c-fos antibodies were from Cell Signaling Technology (Beverly, MA). Anti-β-actin antibody was from Sigma-Aldrich (Lyon, France) and anti-NFATc1 antibody was from Abcam (Cambridge, MA, USA). Anti-CD200-APC antibody was from Ebioscience (San Diego, CA, USA). Recombinant human CD200 Fc chimera protein (rCD200) was from R&D Systems (Minneapolis, MN, USA).

### Isolation of peripheral blood mononuclear cells (PBMCs)

Human PBMCs were obtained from normal blood-donor buffy coats (kindly provided by Etablissement Français du Sang Centre Atlantique and Pyrénéees Méditerranée, France) after density gradient centrifugation. Briefly, buffy coat was diluted 1:1 in phosphate buffered saline (PBS), layered over Ficoll-Hypaque (Eurobio, Courtaboeuf, France) and centrifuged at 600g for 30 min. The interface layer was washed twice in PBS and re-suspended in alpha-modified Eagle’s medium (α-MEM) supplemented with 2 mM L-glutamine, 100 U/mL penicillin, 100 U/mL streptomycin + 10% heat-inactivated fetal calf serum (FCS; Hyclone research grade, Perbio, Brebières, France) (α-MEM/FCS).

### Osteoclast formation

1×10^6^ PBMCs were plated in 96-well plates with α-MEM + 10% FCS (α-MEM/FCS). Two hours later, cells were rinsed with medium to remove non-adherent cells, then cultivated for 2 days in α-MEM/FCS supplemented with M-CSF (50 ng/mL) to generate osteoclast precursors. Osteoclast precursors were further stimulated with M-CSF (50 ng/mL) and RANKL (50 ng/mL) in α-MEM/FCS. To assess the effect of CD200 on osteoclastogenesis, 1 µg/mL of rCD200 was added with M-CSF and RANKL. Freshly made medium, supplemented with full concentrations of cytokines and recombinant proteins, was changed every 3 days, and cultures were maintained for up to 21 days. PBMCs in α-MEM alone represented the negative control. On day 21, cells were fixed and stained for TRAP by use of the acid-phosphatase leucocyte diagnostic kit (Sigma-Aldrich, Lyon, France). Osteoclasts were counterstained with hematoxylin and DAPI, and multinucleated TRAP-positive osteoclasts (3 nuclei and more) were counted in triplicate wells under a light microscope. The area of osteoclasts was determined by use of ImageJ (NIH, Bethesda, MD, USA). For detecting actin ring formation, cells were fixed with 4% paraformaldehyde, permeabilized and incubated with phalloidin-Alexa 540 (Invitrogen, Carlsbad, CA, USA) for 30 min at 4° C. After washing with PBS, nuclei were counterstained with DAPI. Cells were imaged using a microscope with an attached digital camera (Nikon, Champigny sur Marne, France).

To determine the functionality of osteoclasts, 1x10^6^ PBMCs were plated on bone slices (Immunodiagnostic Systems, Paris, France) in wells of 96-well plates as for osteoclast formation. After 21 days of culture, bone slices were washed with PBS and placed in a trypsin solution (0.25 M) for 15 min at 37° C and in a solution of NH_4_OH (1 N) for 30 min to remove all adherent cells. The slices were rinsed in distilled water and stained with 0.1% toluidine blue. The presence of lacunar resorption pits at the bone surface was determined by light microscopy and the area of the pit was measured by use of ImageJ.

To measure the effect of MSCs and specifically the role of CD200 on osteoclast formation, 1x10^4^ MSCs were co-cultivated with PBMCs as described previously.

### MSC isolation and amplification

15-20 mL of bone marrow was harvested by iliac-crest aspiration according to the Ethics Committee of Tours University Hospital. Nucleated bone-marrow cells were seeded on culture flasks at 5×10^4^ cells per cm^2^ and MSCs were isolated by their adherence capacity. Cells were then amplified in α-MEM supplemented with 2 mM L-glutamine, 100 U/mL penicillin, 100 U/mL streptomycin, 10% FCS. Medium was renewed twice a week until cells reached confluence. Cells were harvested from plastic by 25-min incubation with 0.25% collagenase NB4 (Serva Electrophoresis, Heidelberg, Germany).

### CD200^+^/CD200^–^ MSC separation

MSCs were washed 3 times with PBS, detached with collagenase, then incubated in blocking buffer (PBS, 0.5% bovine serum albumin [BSA], 2 mM NaN_3_, 2 mM EDTA, 5% goat serum, 5% rabbit serum) for 30 min at 4° C. Cells were incubated with anti-CD200-APC antibody (eBioscience, San Diego, CA, USA) for 1 hr at 4° C in blocking buffer and washed 3 times with wash buffer (PBS 0.5% BSA, 2 mM NaN_3_, 2 mM EDTA). Then MSCs were probed with anti APC-magnetic microbeads (Miltenyi, GmBH, Germany) for 25 min at 4° C in blocking buffer and washed 3 times. Cell suspension was applied onto an LD column (Miltenyi, GmBH, Germany) and submitted to magnetic separation. The effluent of the column represents the CD200-negative population (CD200^–^ MSCs). The CD200-positive cells (CD200^+^ MSCs) were flushed out of the column, and to increase the purity, the fraction was enriched over a large cell column (Miltenyi, GmBH, Germany) and submitted to additional magnetic separation. The purity of each fraction was determined by flow cytometry.

### Flow cytometry

PBMC adherent cells were stimulated for different times with M-CSF (50 ng/mL), M-CSF + RANKL (50 ng/mL each) with or without recombinant CD200 (rCD200; 1 µg/mL). Cells were washed 3 times with PBS, detached with PBS/EDTA/lidocaine, then incubated in blocking buffer for 30 min at 4° C. Cells were incubated with directly conjugated antibodies for 1 hr at 4° C in blocking buffer and washed 3 times with wash buffer. Cells were fixed with 2% paraformaldehyde, and fluorescence was analyzed on a FACSCalibur cytometer (BD Biosciences, Le Pont de Claix, France) with CellQuest software.

### Western blot analysis

PBMC adherent cells (2×10^6^ cells/well) were cultivated in 6-well plates, activated for 48 hr with M-CSF (50 ng/mL) with or without rCD200 (1 µg/mL) and stimulated for different times with RANKL (50 ng/mL). To prepare whole-cell lysates, cells were washed with PBS and lysed with ice-cold RIPA buffer (Cell Signaling Technology, Beverly, CA, USA) containing 1 mM phenylmethanesulfonyl fluoride (PMSF) and protease inhibitor cocktail (Roche Applied Science, Penzberg, Germany). Twenty micrograms of protein were separated on 6% or 10% SDS-PAGE and transferred to PVDF membrane (BioRad, Marnes-la-Coquette, France). The membrane was blocked in 5% milk in PBS/0.1% Tween-20 and probed overnight with the primary antibodies for ERK1/2, phospho-ERK, p38, phospho-p38, c-fos (Cell Signaling Technology, Beverly, CA, USA), NFATc1 (Abcam, Cambridge, MA, USA) and β-actin (Sigma-Aldrich, Lyon, France). After a washing with PBS/Tween, blots were probed with horseradish peroxidase-conjugated secondary antibody (BioRad, Marnes-la-Coquette, France) for 1 hr at room temperature. Staining was detected by chemiluminescence (ECL protein detection system; GE Healthcare, Europe, Velizy-Villacoublay, France), and bands were visualized by use of ChemiDoc (BioRad, Marnes-la-Coquette, France).

### Gene expression analysis

Total RNA was extracted by use of the RNeasy midi kit (Qiagen, Courtaboeuf, France) and 1 µg RNA was reverse transcripted by use of the Prime Script RT-PCR kit (Takara Biotechnology, Mountain View, CA, USA). Real-time quantitative PCR involved use of Sso Fast™ Eva Green® supermix (BioRad, Marnes- la- Coquette, France) and the CFX 96 thermocycler (BioRad, Marnes- la- Coquette, France). Gene expression was normalized relative to GAPDH expression. The primer sequences were for TRAP, forward, 5’-AAG-ACT-CAC-TGG-GTG-GCT-TTG-3’ and reverse, 5’-GGC-AGT-CAT-GGG-AGT-TCA-GG; NFATc1, forward, 5’-GCA-TCA-CAG-GGA-AGA-CCG-TGT-C-3’ and reverse, GAA-GTT-CAA-TGT-CGG-AGT-TTC-TGA-G-3’; CTSK, forward, 5’-GCC-AGA-CAA-CAG-ATT-TCC-ATC-3’ and reverse, 5’-CAG-AGC-AAA-GCT-CAC-CAG-AG-3’; and GAPDH, forward, 5’-GCC-CAA-TAC-GAC-CAA-ATC-C-3’ and reverse, 5’-AGC-CAC-ATC-GCT-CAG-ACA-3’ (IDT, Leuven, Belgium.) 

### Statistic analysis

Data are reported as mean ± SD using GraphPad Prism Software (GraphPad Softaware, San Diago, CA, USA). One-tailed Mann-Whitney test was used.

## Results

### Recombinant CD200 inhibits osteoclast formation

**Figure 1:**
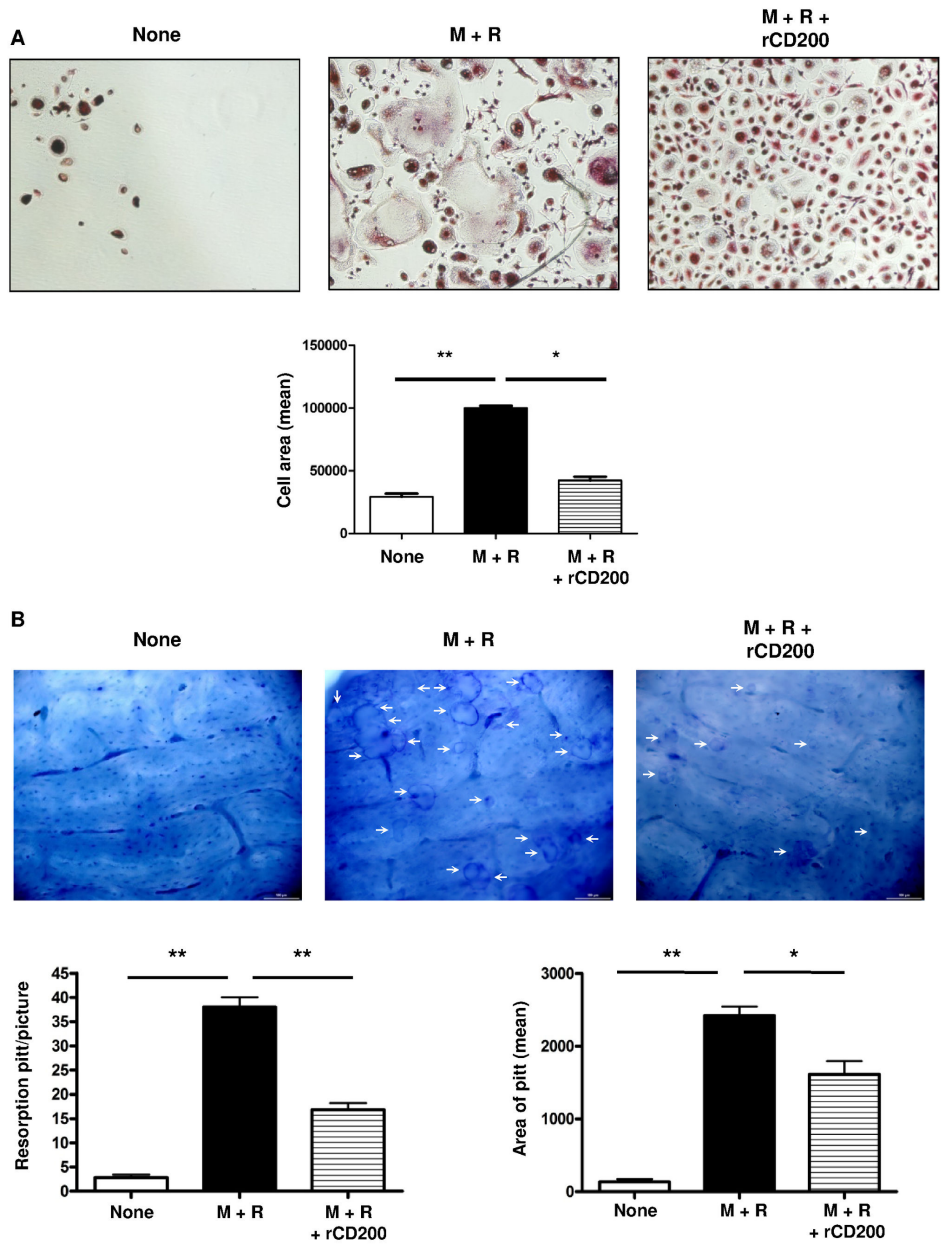
Recombinant CD200 inhibits osteoclast formation. (A) Adherent peripheral blood mononuclear cells (PBMCs) were cultured with M-CSF for 48 hr, RANKL was added on day 3 of culture and medium was changed every 3 days. rCD200 was added at the beginning of the culture and every 3 days. After 21 days of culture, osteoclast formation was determined by the use of tartrate-resistant acid phosphatase (TRAP) assay. Data are mean±SD area of osteoclasts from 3 independent experiments. (B) rCD200 inhibits bone resorption activity. PBMCs were plated on bone slices in wells of 96-well plates and stimulated as described. After 21 days of culture, resorbtion pitt number and area were count under a light microscope (arrows). **, P<0.01 compared with none; *, P<0.05 compared with M+R alone.

The CD200–CD200R interaction can deliver an inhibitory signal to monocytes/macrophages [18,19]. To determine the effect of CD200 on osteoclastogenesis, we used a validated culture system: PBMCs were cultivated for 2 days with M-CSF to induce RANK expression and then treated with RANKL + M-CSF for 21 days to induce the formation of multinucleated TRAP+ osteoclast-like cells capable of resorbing bone surface (Figure 1A,B). The addition of different concentrations of rCD200 to the culture medium inhibited the RANKL-induced formation of osteoclasts (Figure 1A and Figure S1) and decreased the size and number of resorption pits on the bone surface (Figure 1B). Thus, rCD200 inhibited osteoclast formation and decreased their bone degradation capacity.

### Recombinant CD200 does not modify the phenotype of osteoclast precursors

**Figure 2:**
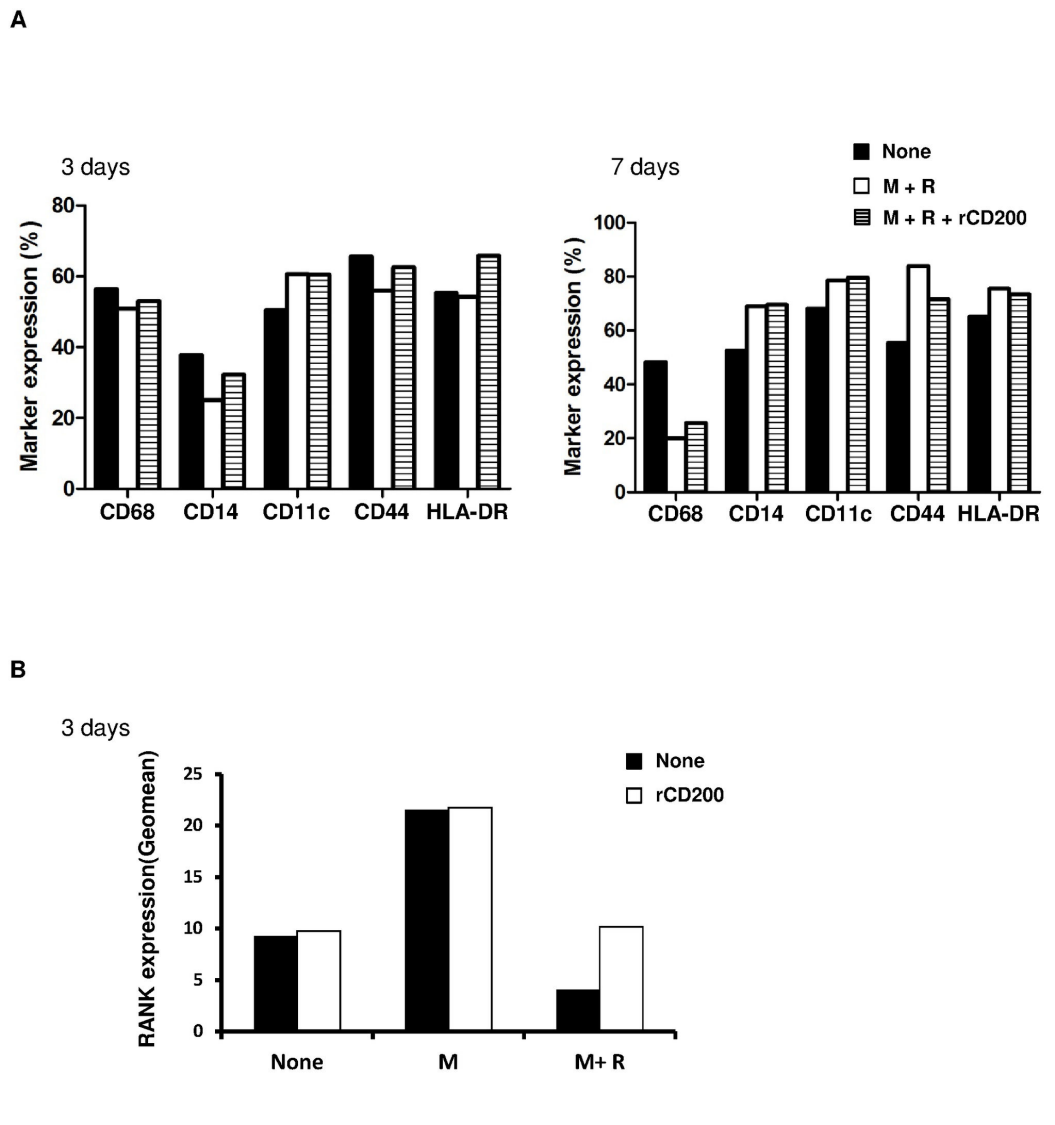
rCD200 does not modify osteoclast precursor phenotype but increase RANK receptor at the cell surface. PBMC adherent cells were cultivated with M-CSF or M-CSF + RANKL for 3 or 7 days with or without rCD200. The expression of surface markers (A) and RANK (B) was determined by flow cytometry. One experiment representative of 2 is shown.

The inhibitory effect of CD200 on osteoclast formation could be associated with a modified phenotype of monocytes/macrophages. PBMC adherent cells were treated for 3 or 7 days with M-CSF + RANKL with or without rCD200. The presence of rCD200 did not modify the phenotype of monocytes after 3 or 7 days of treatment compare to M-CSF + RANKL alone (Figure 2A).

The induction of osteoclast formation is associated with the expression of RANK at the cell surface of monocytes. As expected, M-CSF increased the expression of RANK and co-treatment with M-CSF + RANKL inhibited this expression after 3 days. rCD200 did not change RANK expression induced by M-CSF but surprisingly limited the decrease in RANK expression induced by RANKL (Figure 2B). Therefore, rCD200 inhibited osteoclast formation without modifying the phenotype of osteoclast precursors but surprisingly increased RANK expression.

### Recombinant CD200 inhibits the RANKL-induced MAPK signaling pathway

**Figure 3:**
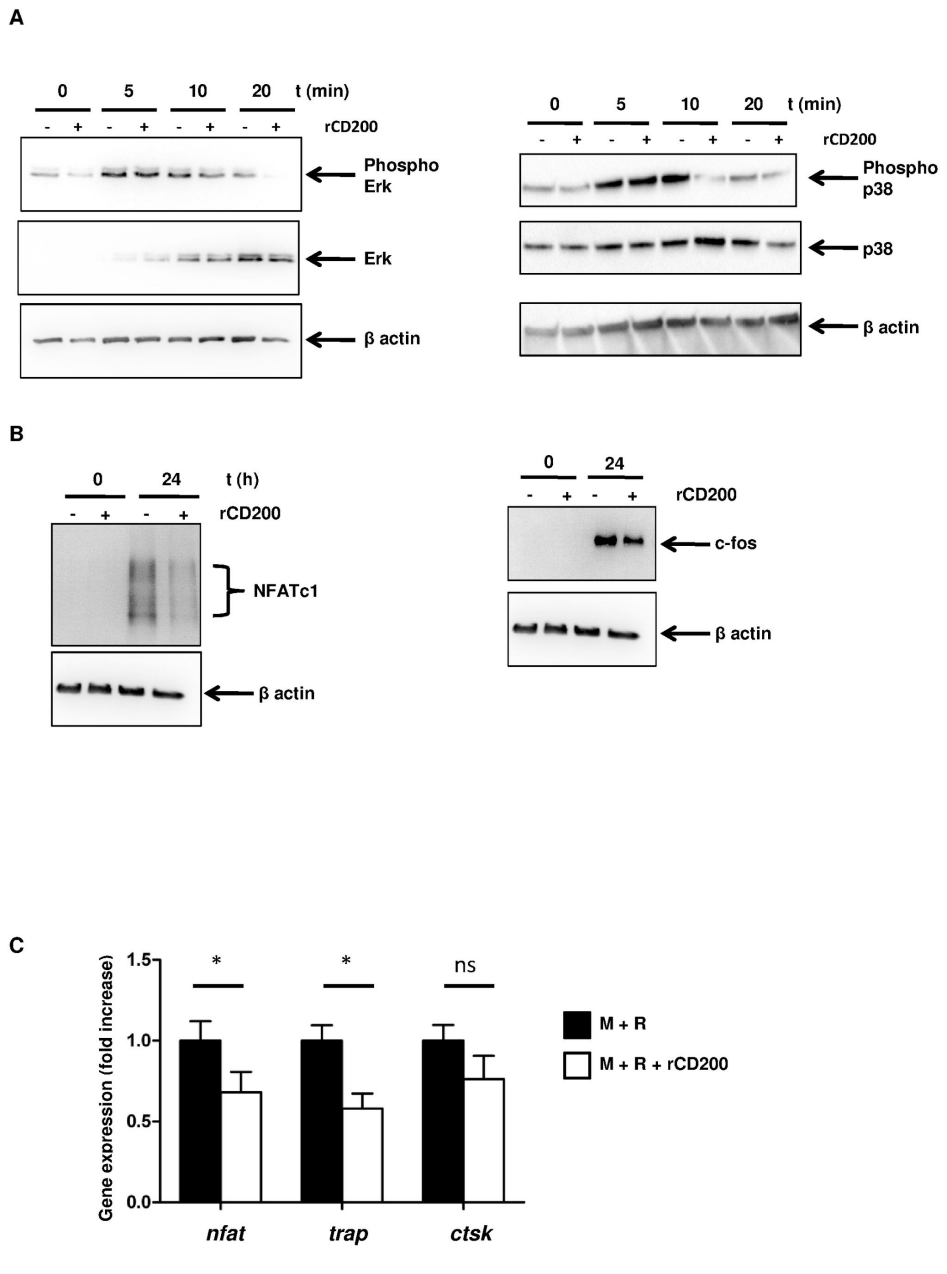
rCD200 inhibits RANKL signaling pathway. (A and B) PBMC adherent cells were cultured with M-CSF with or without rCD200 for 48 hr and stimulated for different times with RANKL. Expression of total protein levels and of the phosphorylated forms of p38, ERK, c-fos and NFATc1 was determined by Western blotting. The results of 1 experiment, representative of 3, are presented. (C) rCD200 inhibits osteoclast-related gene expression. Cells were cultivated with M-CSF for 48 hr, RANKL was added on day 3 of culture and medium was changed every 3 days. rCD200 was added at the beginning of the culture and every 3 days. Expression of target gene was determined by real-time PCR after 21 days of culture. Data are mean±SD from 3 independent experiments. *, P<0.05 compared with M+R alone, ns: non significant.

We next determined the mechanism by which CD200 inhibits osteoclast formation. Osteoclastogenesis depends on the activation of different signaling pathways induced by RANKL, so we investigated the effect of rCD200 on RANK signaling. PBMC adherent cells were treated for 48 hr with M-CSF with or without rCD200 and stimulated for different times with RANKL. RANKL induced a rapid phosphorylation of ERK and p38; in presence of CD200 the phosphorylation of p38 and Erk rapidly decreased compare to RANKL induction (Figure 3A). The induction of signaling pathways by RANK is dependent on RANKL-induced MAPK pathway. We therefore determined whether rCD200 modulated the expression of two essential osteoclastogenic transcription factors, NFATc1 and c-Fos [5,20]. As expected, within 24 hr culture, RANKL induced NFATc1 and c-fos protein expression, and rCD200 pretreatment abrogated this induction (Figure 3B). Moreover, CD200 inhibited significantly the expression of RANKL-induced osteoclast-associated genes *trap* (p=0.029), and *nfatc1* (p=0.014) but not *cathepsin k* (Figure 3C). Therefore, rCD200 inhibited osteoclastogenesis by inhibiting the MAPK pathway.

### CD200^+^ population in MSCs inhibits osteoclast formation

**Figure 4:**
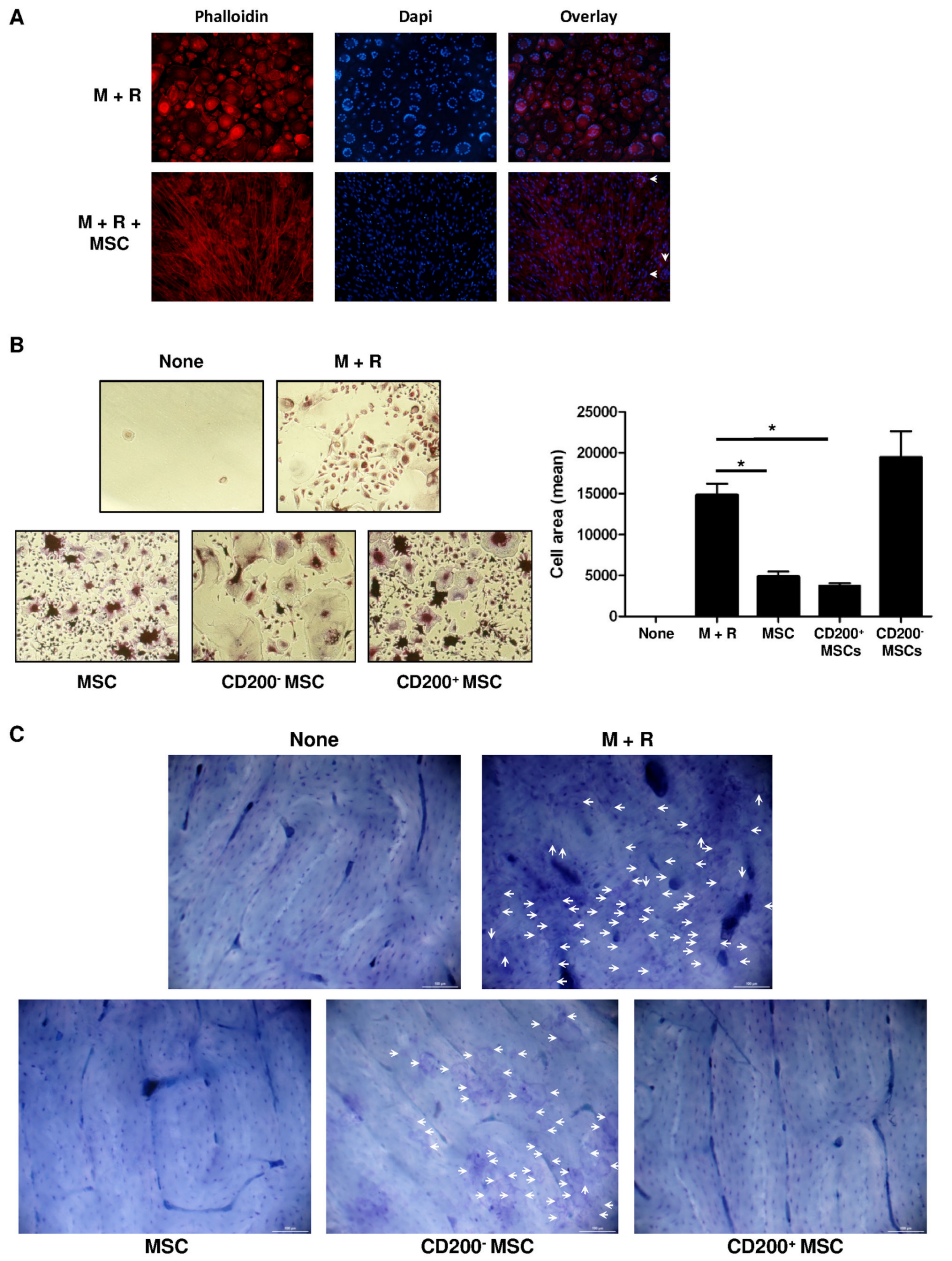
Within MSCs, CD200^+^ population inhibits osteoclast formation. PBMC adherent cells were co-cultured with MSCs in presence of M-CSF for 48 hr. RANKL was added after 48 hr and half of the medium was changed every 3 days. (A) After 21 days of culture, actin rings were stained with phalloidin-Alexa 540 and nuclei with DAPI. (B) Osteoclast precursors were cultivated with MSCs, CD200^+^ MSCs or CD200^–^ MSCs for 21 days. Tartrate-resistant acid phosphatase (TRAP) assay was performed and area of TRAP-positive cells was measured. Data are mean±SD. *, P<0.05. (C) Effect of MSCs on bone resorption activity of osteoclasts. White arrows represent bone resorption pits. The results of 1 experiment out of 3 are presented.

As previously described, CD200 is expressed at the cell surface of a population of MSCs [17]. However, few studies have investigated the effect of MSCs on human osteoclastogenesis [21]. So, we next determined the effect of MSCs on osteoclast formation and more precisely the role of CD200, expressed on the cell surface of a population of MSCs. PBMC adherent cells were cultivated with human MSCs (1×10^4^ cells per well) in α-MEM + M-CSF for 48 hr and then cultivated in differentiation medium (M-CSF + RANKL) for 21 days. Cells cultivated with M-CSF + RANKL differentiated into multinuclear cells that formed peripheral actin ring, a marker of a late stage of osteoclast differentiation. Co-culture with MSCs induced a drastic inhibition of RANKL-dependent osteoclast formation characterized by decreased multinucleated cell number and presence of mononuclear cells with diffuse actin staining (Figure 4A). A subpopulation of MSCs expressed CD200 at the cell surface. We magnetically separated CD200^+^ MSCs from CD200^–^ MSCs and isolated two pure fractions (Figure S2). Each fraction was cultivated with PBMC adherent cells in differentiation medium for 21 days. As expected, co-culture of PBMC adherent cells with MSCs inhibited the formation of TRAP+ multinucleated cells. Interestingly, inhibition of osteoclast formation differed by MSC fraction (Figure 4B). Co-culture with the CD200^+^ fraction inhibited osteoclast activity, characterized by the absence of resorption pits at the bone surface, whereas co-culture with CD200^–^ MSCs did not modify osteoclast function (Figure 4C). Moreover, we demonstrated that CD200^-^ MSCs and CD200^+^ MSC expressed a similar level of OPG (Figure S3) confirming that the inhibitory effect of CD200^+^ fraction is independent on OPG secretion. Therefore, MSCs, via expression of CD200 at the cell surface, inhibited osteoclast formation.

## Discussion

Our study demonstrates that CD200 is a key regulatory molecule of osteoclastogenesis (Figure 1) and that MSCs, via CD200 expression at their cell surface, block osteoclast formation and their bone-degradation capacity (Figure 4) by inhibiting the downstream RANK signaling pathway (Figure 3).

Previous papers demonstrated that the CD200–CD200R1 axis is one of the regulatory mechanisms of myeloid lineage functions. CD200, widely expressed on many cell types, transmits an inhibitory signal in macrophages through CD200R1 and represses macrophage commitment and differentiation [14,18,22]. *In vivo*, inhibition of CD200R induced hyperactivation of macrophages and development of autoimmune disease [11]. We used recombinant CD200Fc fusion protein but it mechanism of action is still under questions. Hatherley et al demonstrated that the extracellular domain of mouse CD200Fc fusion protein did not bind to the CD200R [23]. Gorkcynski et al showed that CD200Fc modulated DC activity but to a lesser extent than endogenous CD200 from transgenic mice. The difference of effect could be linked to a lower binding affinity of soluble CD200Fc protein for all CD200Rs than the natural cell surface CD200 (endogenous or the transgene) or that the CD200 expressed at the cell surface present a different ligand domain compare to the soluble protein [24]. However, the effect of CD200Fc chimera protein was demonstrated in different *in vivo* studies [25,26]. In our experiment, similar effects on osteoclast formation are obtained with both soluble CD200Fc protein and the CD200 expressed at the MSC surface.

We demonstrated that soluble CD200 or that expressed at the MSC surface inhibited the RANK signaling pathway but increased the expression of RANK at the cell surface of osteoclast precursors. Little is known about RANK receptor degradation and recycling, but Cbl-b protein was found to promote ubiquitin-mediated proteasome degradation as well as RANK recycling at the membrane level [27].

Interestingly, a recent paper demonstrated that CD200^+^ MSCs inhibited the production of TNF-α and modulated the immune response of macrophages [28]. MSCs modulate the differentiation of myeloid cells such as dendritic cells [29,30] and monocytes [31]. MSCs interfere with the polarization of macrophages, turning lipopolysaccharide (LPS)-activated macrophages into a regulatory-like phenotype similar to alternative-activated macrophages characterized by increased production of interleukin 10 and 6 (IL-10 and IL-6) and low secretion of pro-inflammatory cytokines [32,33]. Similarly in mice, MSCs inhibit TNF-α and IL-6 secretion and stimulate IL-10 production and phagocytosis of apoptotic cells by LPS-activated macrophages [34]. Moreover, MSCs impair the activation of microglia, resident macrophages of the central nervous system, thus inducing the expression of neuroprotective molecules such as CD200R [35].

MSCs can modulate macrophage differentiation, but their effect on osteoclast formation has been controversial. Our results show that MSCs inhibit osteoclast formation and confirm previous data in humans [21]. On the contrary, in mice, MSCs promote osteoclast differentiation, which is increased in the presence of M-CSF + RANKL [36]. However, in both species, the mechanism of action of MSC on osteoclastogenesis seems unclear. Oshita et al. showed that MSCs inhibit osteoclastogenesis through OPG secretion, independent on cell–cell contact [21]. In our system, OPG seems to have no role in the negative effect of MSC on osteoclast formation. Indeed, CD200^-^ MSCs and CD200^+^ MSCs expressed a similar level of OPG after 4 days of M-CSF treatment. Moreover, in presence of monocytes + M-CSF, CD200^-^ MSCs seems to produce more OPG than CD200^+^ MSCs (supplementary data: Figure 2) confirming that the inhibitory effect of MSCs on osteoclast formation depends on the CD200 expression.

Nevertheless, other proteins involved in the contact between MSCs and osteoclasts control osteoclast functions. The interaction of ephrinB2, expressed on osteoclasts, and EphB4, expressed on osteoblast precursors, modulates osteoclast activity as well as osteoblast differentiation. A reverse signal, through ephrinB2, suppresses osteoclast function, whereas a forward signal, through EphB4, enhances osteoblast differentiation [37]. Moreover, the interaction between intercellular adhesion molecule 1 (ICAM-1), expressed on the osteoblast surface and lymphocyte function-associated antigen 1 (LFA-1), on osteoclast precursors, promotes differentiation and maturation of osteoclasts [38,39]. Finally, determining the effect of CD200–CD200R interaction on MSC functions is of interest.

The role of the CD200–CD200R axis in osteoclast formation in mice was studied. Therefore Cui et al. demonstrated that CD200 expression is an endogenous signal used by macrophages to control their fusion to form osteoclasts [10]. We demonstrated that in human, CD200 expressed on MSC surface is an exogenous mechanism which inhibits the osteoclast formation. However, in line with our results, Lee et al. showed that soluble CD200, secreted by osteoblasts, inhibited osteoclast formation in mice [9]. These data corroborate our findings and confirm the negative role of CD200 on osteoclast formation through two different pathways: direct contact with CD200R or modulation of soluble factor secretion.

Native MSCs express different levels of CD200 at the cell surface [28], but the localization of each subpopulation in bone has not yet been determined. The possible localization of CD200^+^ MSCs close to osteoclast precursors could confirm the important role of the CD200–CD200R interaction in the control of osteoclast formation.

Taken together, our results demonstrate that through the surface expression of CD200, MSCs play a role in regulating bone resorption and bone physiologic aspects. The CD200–CD200R interaction controls osteoclastogenesis and could be a new target to modulate osteoclast function and control bone pathologies such as osteoporosis.

## Supporting Information

Figure S1 Effect of different concentration of rCD200 on osteoclast formation. After 21 days of culture, osteoclast formation was determined by the use of tartrate-resistant acid phosphatase (TRAP) assay.
(TIF)

Figure S2 CD200 expression of MSC fraction after magnetic separation. CD200 expression of cells from each fraction was determined by flow cytometry.
(TIF)

Figure S3 OPG secretion by CD200^-^ and CD200^+^ MSCs: CD200- and CD200+ MSCs are obtained after magnetic separation and culture 4 days in α-MEM/FCS supplemented with M-CSF (50 ng/mL) in presence (monocyte) or in absence (none) of monocytes. OPG concentration in culture supernatants is determined by ELISA according to the distributor’s instructions (RayBiotech, Norcross, GA, USA). ns: non significant.
(TIF)
