# Supplementary figures and images for: CD200R/CD200 Inhibits Osteoclastogenesis: New Mechanism of Osteoclast Control by Mesenchymal Stem Cells in Human
Source: PLoS One. 2013 Aug 5;8(8):e72831. doi: 10.1371/journal.pone.0072831 (PMC3733817; doi:10.1371/journal.pone.0072831)

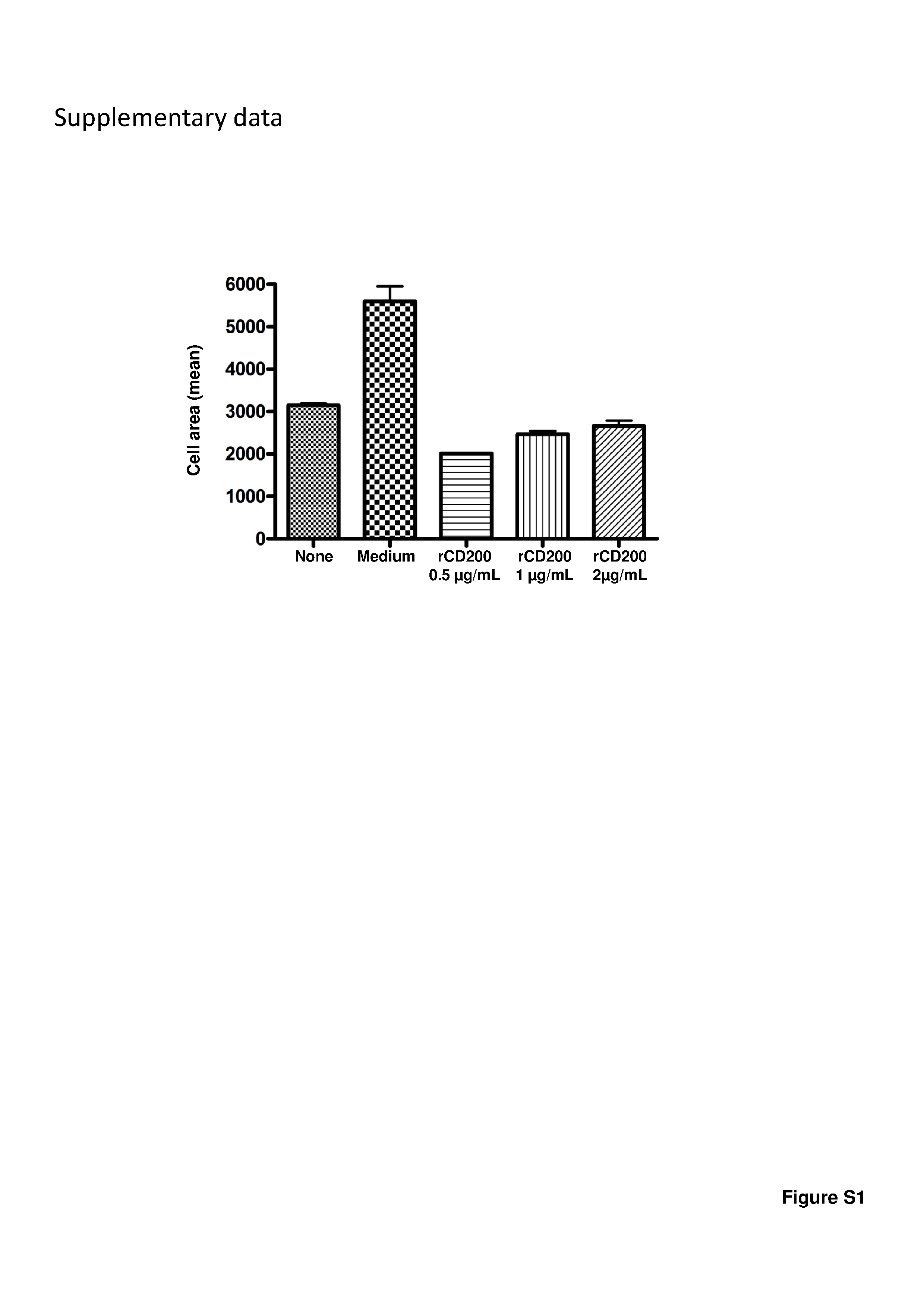

Supplement: Figure S1 — After 21 days of culture, osteoclast formation was determined by the use of tartrate-resistant acid phosphatase (TRAP) assay. (TIF) [file pone.0072831.s001.tif]

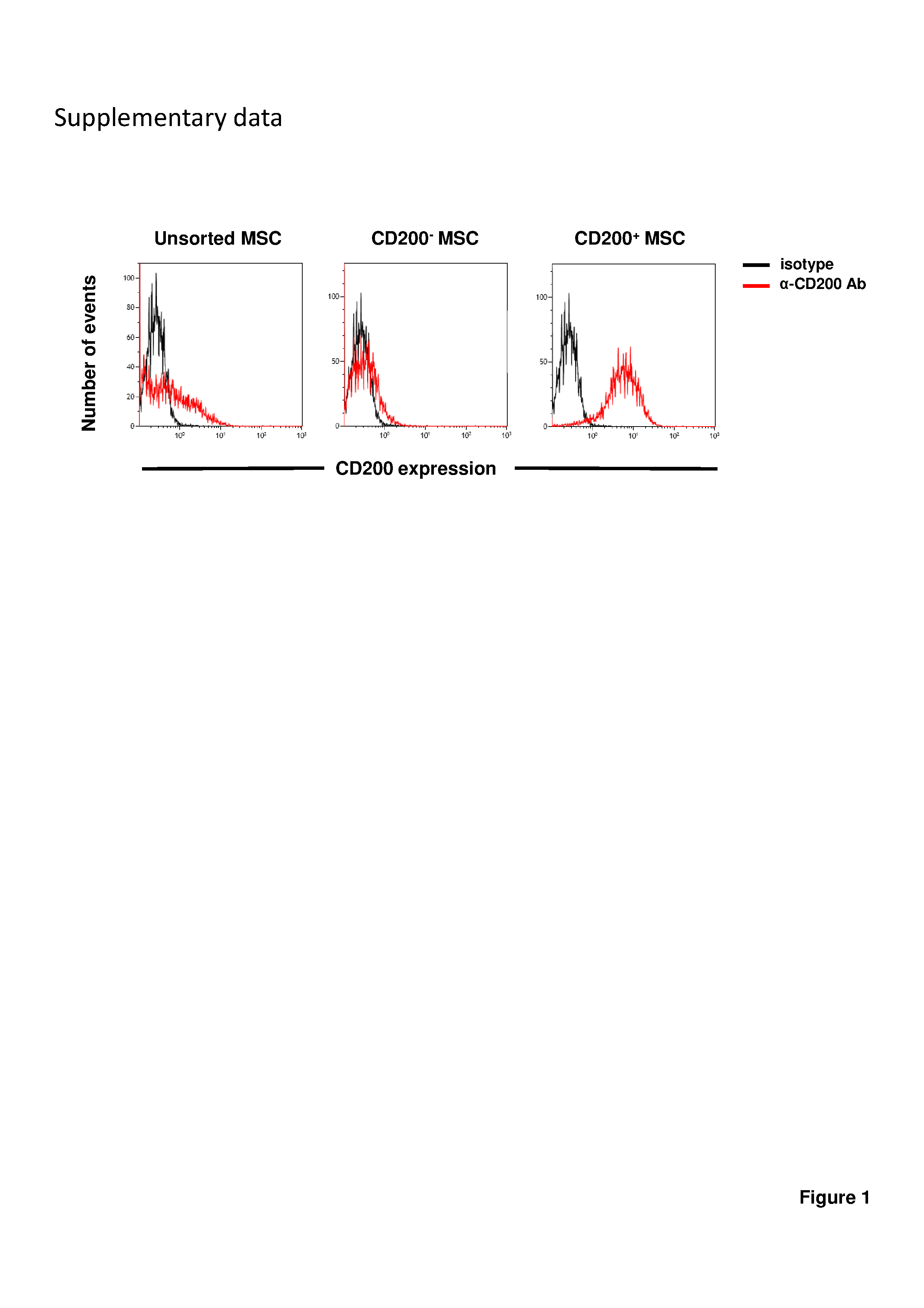

Supplement: Figure S2 — CD200 expression of cells from each fraction was determined by flow cytometry. (TIF) [file pone.0072831.s002.tif]

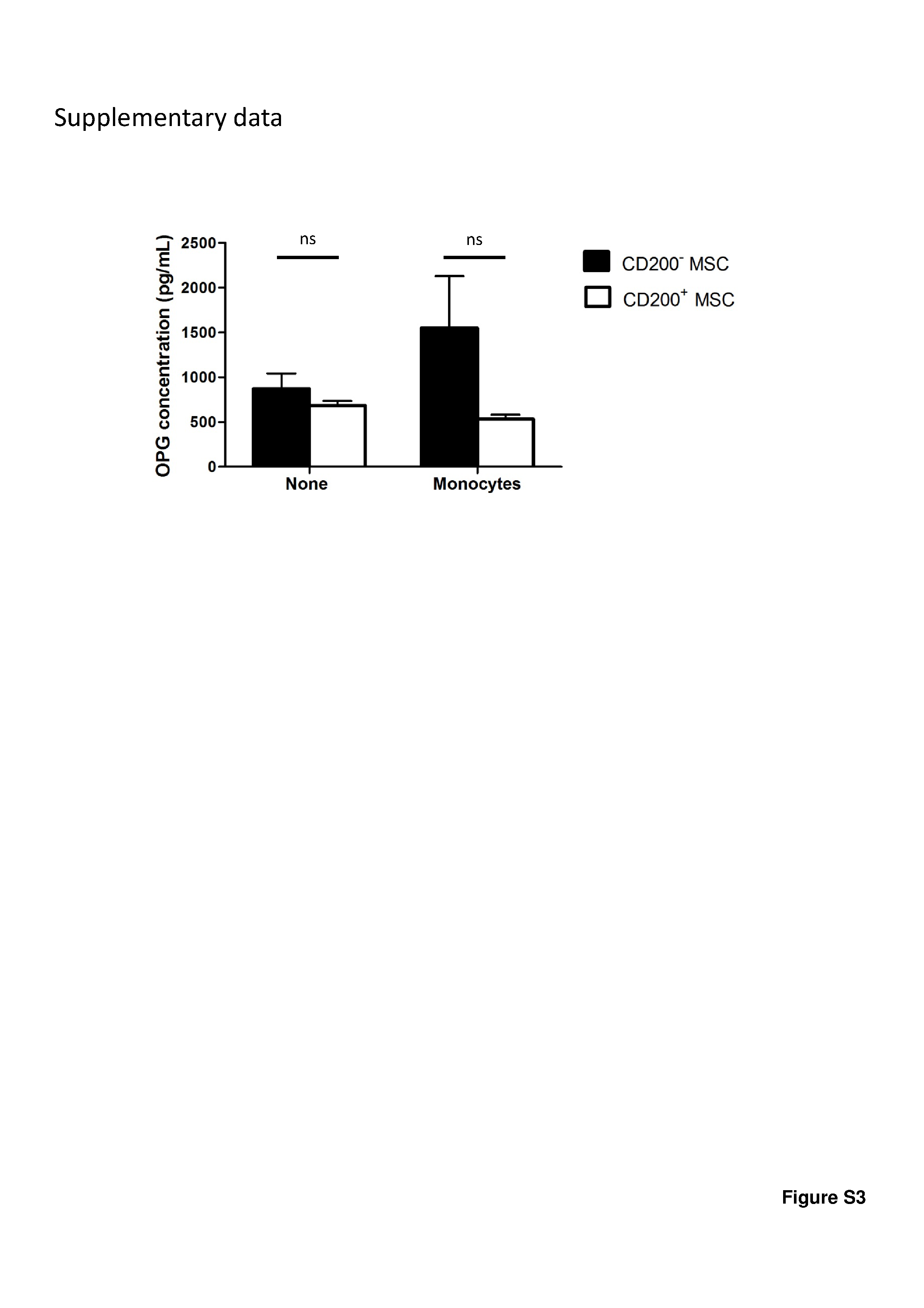

Supplement: Figure S3 — CD200- and CD200+ MSCs are obtained after magnetic separation and culture 4 days in α-MEM/FCS supplemented with M-CSF (50 ng/mL) in presence (monocyte) or in absence (none) of monocytes. OPG concentration in culture supernatants is determined by ELISA according to the distributor’s instructions (RayBiotech, Norcross, GA, USA). ns: non significant. (TIF) [file pone.0072831.s003.tif]
